# Human- Versus Machine Learning–Based Triage Using Digitalized Patient Histories in Primary Care: Comparative Study

**DOI:** 10.2196/18930

**Published:** 2020-09-03

**Authors:** Artin Entezarjou, Anna-Karin Edstedt Bonamy, Simon Benjaminsson, Pawel Herman, Patrik Midlöv

**Affiliations:** 1 Center for Primary Health Care Research Department of Clinical Sciences in Malmö/Family Medicine Lund University Malmö Sweden; 2 Clinical Epidemiology Division Department of Medicine Solna Karolinska Institute Stockholm Sweden; 3 Doctrin AB Stockholm Sweden; 4 Smartera AB Stockholm Sweden; 5 Department of Computational Science and Technology KTH Royal Institute of Technology Stockholm Sweden

**Keywords:** machine learning, artificial intelligence, decision support, primary care, triage

## Abstract

**Background:**

Smartphones have made it possible for patients to digitally report symptoms before physical primary care visits. Using machine learning (ML), these data offer an opportunity to support decisions about the appropriate level of care (triage).

**Objective:**

The purpose of this study was to explore the interrater reliability between human physicians and an automated ML-based triage method.

**Methods:**

After testing several models, a naïve Bayes triage model was created using data from digital medical histories, capable of classifying digital medical history reports as either in need of urgent physical examination or not in need of urgent physical examination. The model was tested on 300 digital medical history reports and classification was compared with the majority vote of an expert panel of 5 primary care physicians (PCPs). Reliability between raters was measured using both Cohen κ (adjusted for chance agreement) and percentage agreement (not adjusted for chance agreement).

**Results:**

Interrater reliability as measured by Cohen κ was 0.17 when comparing the majority vote of the reference group with the model. Agreement was 74% (138/186) for cases judged not in need of urgent physical examination and 42% (38/90) for cases judged to be in need of urgent physical examination. No specific features linked to the model’s triage decision could be identified. Between physicians within the panel, Cohen κ was 0.2. Intrarater reliability when 1 physician retriaged 50 reports resulted in Cohen κ of 0.55.

**Conclusions:**

Low interrater and intrarater agreement in triage decisions among PCPs limits the possibility to use human decisions as a reference for ML to automate triage in primary care.

## Introduction

Health care digitalization has the potential to mitigate increasing primary care workloads [1,2]. Time-constrained primary care physicians (PCPs) interrupt patient queries within the first 30 seconds of consultations [3], contributing to inadequate gathering of medical histories [4,5]. To reduce PCP workload and to ensure patients are directed to the appropriate level of care, nurse-led telephone triage is commonly used [6,7]. However, nurses face similar time constraints as physicians, which results in incomplete gathering of medical histories [8] and inappropriate levels of care recommended in up to 31% of cases [9,10].

Leveraging the wide use of smartphones, a large portion of patient history can today be acquired before the patient interacts with his/her health care provider. Automated patient interviewing software has been shown to gather reliable and relevant clinical information [11], and may thus save clinicians time and reduce workloads.

Existing “symptom checkers” can provide triage recommendations directly to patients. However, their accuracy is low, ranging from 33% to 78%, with higher accuracy reported only for more acute conditions [12]. Furthermore, patient adherence to symptom checker recommendations seems low at just 65% [13], compared with 81%-100% adherence to advice from triage nurses [7]. Thus, clinician decision-support software may be a better solution for optimizing triage.

With rapid developments in machine learning (ML), labeled automated patient interviewing software data offer a promising opportunity for enhancing triage software accuracy, providing appropriate access to primary care. Recent research shows promising utility of ML to aid in emergency department triage compared with commonly used algorithms [14]. However, the performance of such a system compared with human triage has, to the best of our knowledge, never been evaluated. Furthermore, ML research in the primary care setting is lacking, despite over 60% of health care visits being conducted in primary care [15].

Thus, this study sought to investigate interrater reliability between human physicians and an automated ML-based triage method, as well as evaluating interrater reliability of triage decisions between a panel of physicians assessing the same patient histories from an automated patient interviewing software.

## Methods

### Context

The automated patient interviewing software technology used in this study (produced by Doctrin AB, Stockholm, Sweden) is being used by several primary care providers in Sweden since 2017. Patients access the platform using their smartphone, tablet, or computer, choosing their chief complaint from a prespecified list. An automated medical history is then taken, allowing patients to briefly formulate ideas, concerns, and expectations in free-form text, and subsequently answer a symptom-specific multiple-choice survey. The software selects suitable subsequent survey questions based on the patient’s answers (Table 1).

**Table 1:** Examples of automated patient interviewing software survey questions. Chosen answers subsequently appear in reports used for triage.

Survey question	Answer format
“How long have you had a cough?”	Short answer: specify number of days, months or years
“How has your cough been since it started”	Multiple choice (one option allowed): “Not changing” “Getting worse” “Improving” “Gone away”
“Do you have any of the following symptoms?”	Multiple choice (multiple options allowed): “Runny nose” “Shortness of breath” “Chest pain” “Sore throat” “Swollen glands” “Fever”
If a patient reports fever: “What was the highest temperature you have had when you measured it?”	Multiple choice: “37°C” […] “Over 40 C”
“How many days in a row have you had fever?”	Short answer: specify number of days

Answers are presented to a PCP as a summarized report for review and further doctor–patient communication may occur asynchronously through a live text chat (eVisit). Physicians can prescribe medications, order laboratory samples, provide patient information, or remain available online for up to 72 hours for conservative management. Anonymized data from the automated patient interviewing software report and subsequent chat are saved in a database used for this study. Clinical decisions regarding triage and treatment are, however, recorded separately in the patient medical record and were not accessible for study.

### Data for Classification

Data used in this study were composed of 2 subsets. The first subset consisted of 300 automated patient interviewing software reports labeled by a selected expert PCP with over 10 years of clinical experience and a year of experience with online consultations. The reports represented the 10 most common chief complaints in the platform (common cold, cough, eye redness, genital problems, hay fever, rash, headache, sinus symptoms, sore throat, and urinary tract infections) with an equal marginal distribution between chief complaints. Automated patient interviewing software reports were triaged by the expert PCP to one of 4 levels: (1) Start a digital chat-based consultation; (2) Refer the patient to a primary care center for nonurgent care; (3) Refer the patient to a primary care center for urgent care; or (4) Refer the patient to the emergency department.

The second subset was 300 new automated patient interviewing software reports labeled by a panel of 5 PCPs (1 intern [AE], 2 residents, and 2 specialists). Sample sizes were chosen for feasibility reasons. Each PCP individually triaged automated patient interviewing software reports with an identical distribution of chief complaints as in the first subset. Each automated patient interviewing software report was labeled with a triage level as determined by a majority vote by the panel.

Triage categories in both subsets were then dichotomized into 2 triage levels used for further analyses: (1) No need for urgent physical examination (triage levels 1 and 2) or (2) Need of urgent physical examination (triage levels 3 and 4).

### Exclusion Criteria

Because of incorrect formatting of one of the reports in the triage interface used by the panel, 299 automated patient interviewing software reports were triaged instead of 300.

Automated patient interviewing software reports describing cases with an ongoing medical contact or a different chief complaint from the one specified were classified as inappropriate for triage, which occurred in 37 reports classified by at least one panel member. These were manually reviewed by one of the authors (AB) for inclusion or exclusion by expert opinion, resulting in the exclusion of 17 cases from the analysis.

If the panel voting strategy did not result in a majority for 1 triage level, the automated patient interviewing software report was also excluded from the analysis, which occurred in 6 cases.

Initially, 22 automated patient interviewing software reports had missing triage data from some panel members. After applying the exclusion criteria, 16 automated patient interviewing software reports with missing triage data remained for analysis.

### Model Analyses

To examine the potential of our ML-based approach for triage, we used the available data and corresponding dichotomized triage categories in a series of classification tests with 3 classifiers: (1) a simple linear naïve Bayes classifier, which assumes statistical independence of input features; (2) logistic regression, commonly used for binary classification problems; and (3) random forest, an ensemble decision tree approach, which is considered particularly suitable for high-dimensional problems.

Because of many questions from the automated patient interviewing software reports only appearing very rarely in the small-sized training data, feature space was reduced by only including those which were used in more than 5% of the training samples. This resulted in 243 features. As a few fields included brief free-form text, the classifiers were trained and tested both with and without information extracted from these text data. Text was handled by first removing common Swedish stop words. The remaining commonly used words appearing in more than 10% of the training samples were included as a bag-of-words model where each word was treated as an input feature to the classifier [16]. This resulted in a total of 53 features.

First, we trained the models on the first subset and tested them in a single pass on the second subset with labels based on the majority vote of the 5 PCPs. We complemented this analysis with a cross-validation approach on the data without text information to better estimate generalization capabilities across the 2 subsets of data. We performed 10-fold cross-validation by dividing the union of the 2 subsets into 10 data clusters, where the mixture of the 2 subsets in 9 out of 10 clusters was used for training and the remaining cluster accounting for 10% (ie, 1/10) served as a test set. By applying this scheme 10 times with different 10% test folds, we could obtain an estimate of the second moment of the generalization classification performance. The cross-validation results were followed up with a nonparametric Friedman test.

We made an attempt at investigating the key input features that had a decisive role in classification. To this end, we ranked the coefficients in the regression models built using naïve Bayes and logistic regression methods as well as variable importance with a random forest approach [17]. We employed the correlation of rank, Kendall τ estimator, to examine the consistency of feature ranking produced by the 3 classifiers:

τ = [(n_c_ – n_d_)]/[n(n – 1)/2]

where n is the number of features, n_c_ is the number of concordant feature pairs, and n_d_ is the number of discordant feature pairs. The pairwise relation between feature pairs (f_i_, g_i_) and (f_j_, g_j_) is considered as concordant if the ranking order between features f is the same as for features g, that is, rank (f_i_,) > rank (f_j_,) and rank (g_i_,) > rank (g_j_,), or rank (f_i_,) < rank (f_j_,), and rank (g_i_,) < rank (g_j_,). If neither of these relation pairs is preserved, feature pairs are referred to as discordant.

Finally, in order to exploit diagnostic evaluation made by each individual PCP in the second data subset, rather than directly considering the majority vote as the data sample label, we built 5 independent naïve Bayes classifiers. Each one of them was trained on labels from the second subset corresponding to 1 of the 5 panel PCPs. We then evaluated the majority vote of the dichotomized responses of individual classifiers and employed a cross-validation scheme to estimate generalization properties.

### Human Versus Model Analysis

To measure the agreement between the PCPs and a classification model, we chose a naïve Bayes approach (referred to as “the model”). Cohen κ [18] was calculated to evaluate interrater reliability of triage level within the panel, as well as interrater reliability between the model results and the panel:

κ = (p_o_ – p_e_)/(1 – p_e_)

where p_o_ is the observed ratio of agreement between 2 raters and p_e_ is the probability of chance agreement. Cohen κ provides a measure of agreement between raters while accounting for chance agreements. This is in contrast to percentage agreement, which merely quantifies the ratio of cases with the same classification in relation to different classifications made by 2 or more assessors, without accounting for chance agreements. A Cohen κ<0.20 is generally regarded as low, 0.21-0.40 as fair, 0.41-0.60 as moderate, 0.61-0.80 as substantial, and 0.81-1.00 as almost perfect agreement [18].

### Additional Analyses

To explore how the brief free-form text influenced the classification, the classifier was retrained without features extracted from the brief free-form text. This analysis was conducted with a linear naïve Bayes approach.

To evaluate intrarater reliability of the training data, 50 of the 300 automated patient interviewing software reports available were chosen for retriage by the same expert PCP. These reports were chosen randomly from the full set but checked to include an even variation of all available symptoms. Cohen κ was used to assess agreement with prior triage.

Furthermore, to evaluate the impact of missing data on our results, we reran the analyses with automated patient interviewing software reports with missing triage data excluded.

### Ethical Considerations

The study was approved by the Swedish Ethical Review Authority on April 24, 2019 (reference number 2019-01516).

### Data Sharing Statement

Data on triage decisions made by panel members and our expert PCP are available to the Department of Clinical Sciences in Malmö at Lund university, to the Department of Computational Science and Technology at the Royal Institute of Technology, and to Doctrin AB, Stockholm Sweden 10 years following publication. Data can be accessed for a prespecified purpose after approval by all 3 parties above.

## Results

### Comparisons Between the Three Models

**Figure 1:**
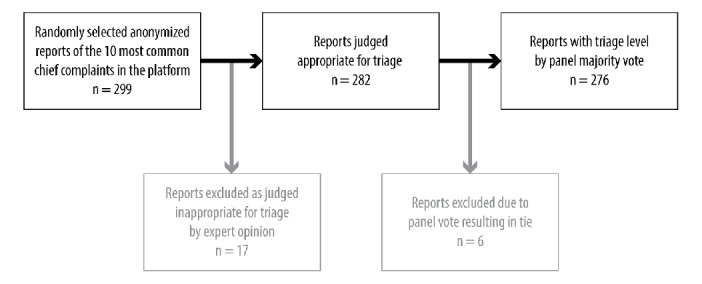
Flowchart of automated patient interviewing software report exclusion criteria.

After exclusion, 276 automated patient interviewing software reports were usable as labeled test-set data (Figure 1). The single-pass test results as well as cross-validation outcomes are presented in Table 2. There was no evidence for rejecting the null hypothesis (*P*>.10), so the performance of all 3 classifiers is considered comparable even though one can observe a trend favorable for random forest.

**Table 2:** Classification results obtained with naïve Bayes, logistic regression, and random forest in a single-pass test as well as in 10-fold cross-validation over the entire combined data set.

Classifier	Test results (training on the first and test on the second data subset), %	10-fold cross-validation (the first and second subsets combined), %^a^
Naïve Bayes	64.1	66.6 (7.6)
Logistic regression	60.1	64.5 (9.0)
Random forest	67.4	69.5 (7.7)

^a^The values for cross-validation are the mean and standard deviation of the classification accuracy obtained over 10 test folds.

### Five Classifiers Versus One

Mean cross-validation accuracy calculated using the ensemble performance (majority vote) of the 5 naïve Bayes classifiers, each trained on the labels of one panel member, was 65.3% (SD 8.2%). Comparing this with the model, that is, the single naïve Bayes classifier (mean cross-validation accuracy 66.7% [SD 8.0%]), the null hypothesis could not be rejected (Wilcoxon signed-rank test, n=10, *P*>.24).

### Decisive Features for Classification

Because the 3 classification approaches offer insights into feature weighing in the regression function that determines the classification boundary, we investigated more closely the distribution of such feature importance factors (see the “Methods” section). The results are inconclusive as the distribution is rather uniform and the pairwise correlations between feature rankings, Kendell τ (see the “Methods” section), produced by the classifiers are moderate (max 0.32 between naïve Bayes and random forest). This result implies that the given average level of accuracy can be achieved based on different sets of features.

### Agreement Between Model and Human Triage

Because there was no statistically significant difference in the performance reported by the 3 classifiers, we decided to rely on the naïve Bayes approach in the next stages of our work due to its intuitive linear formulation. Cohen κ between the naïve Bayes model and the panel majority vote triage was 0.17 (Table 3), with 64% agreement. Excluding the information contained in brief free-form text resulted in the corresponding Cohen κ of 0.15. Within the reference group, average Cohen κ was 0.20, ranging from 0.10 to 0.30.

These results did not differ when analyses were rerun with missing cases excluded. No statistically significant difference in distribution of chief complaint symptoms could be found between reports with and without missing data (chi-square test, *P*>.99).

Using panel majority vote as the gold standard, the model correctly classified 74% (138/186) of nonurgent cases, but only 42% (38/90) of urgent cases. Adding free-form text data had a negligible effect on these numbers (Table 4).

When 50 automated patient interviewing software reports were selected for retriage by our selected expert PCP, Cohen κ was 0.55 with 78% agreement between retriage and previous triage.

**Table 3:** Assessment of the triage performance: agreement between the naïve Bayes model and each panel member as well as their majority vote, and average interrater agreement among the panel members.^a^

Panel	Panel member versus naïve Bayes model (Cohen κ)	Panel member versus rest panel members (Cohen κ)
PCP1	0.09	0.21
PCP2	0.03	0.21
PCP3	0.24	0.18
PCP4	0.08	0.21
PCP5	0.13	0.17
Majority vote	0.17	N/A

^a^PCP1 had the least amount of clinical experience, whereas PCP4 and PCP5 had the most amount of clinical experience.

**Table 4:** Contingency table of model triage with panel majority vote as the gold standard.

	Truly urgent	Falsely nonurgent	Truly nonurgent	Falsely urgent
Naïve Bayes model trained on full information including brief free-form text	42% (38 out of 90 cases voted urgent)	58% (52 out of 90 cases voted urgent)	74% (138 out of 186 cases voted nonurgent)	26% (48 out of 186 cases voted nonurgent)
Naïve Bayes model trained with brief free-form text information excluded	42% (38 out of 90 cases voted urgent)	58% (52 out of 90 cases voted urgent)	73% (135 out of 186 cases voted nonurgent)	27% (51 out of 186 cases voted nonurgent)

## Discussion

### Principal Results

To our knowledge, this is the first study to evaluate human versus ML performance in primary care triage based on a digitalized patient history. The first principal finding of this investigation was that interrater reliability in human triage using automated patient interviewing software reports is low (Cohen κ 0.20). Consequently, our second principal finding was that interrater triage reliability between a statistical model trained on automated patient interviewing software reports and a human panel was low (Cohen κ 0.17).

Findings were robust when cases with missing triage data were excluded from the analysis. The performance of the model was mostly decided by the surveys as removing the free-form text had only marginal impact on Cohen κ (reduced to 0.15). Furthermore, the intrarater reliability was moderate, as seen by retriage of 50 automated patient interviewing software reports by the same PCP (Cohen κ 0.55).

### Comparison With Prior Work

While we acknowledge that κ values seldom are comparable across studies [19], previous data have generally found high interrater reliability between triage nurses [20-22]. However, these studies were conducted in high-acuity emergency department settings, where indicators of urgency arguably are more clearly defined [23].

The primary care setting presents a particular challenge in that conditions are of low acuity, making the line between urgent and nonurgent care more difficult to draw. This is supported by the low intrarater agreement for our expert PCP as well as the low agreement between our panel members. Indeed, acquiring a true gold standard for triage is a well-known issue [24]. “Correct” triage is difficult to define, and thus difficult to label and automate using ML. We could not identify any particular features in the data that were linked to the model’s triage decision. As far as the clinicians are concerned, we did not study their clinical reasoning before reaching a triage decision, that is, we do not know on which features their decision was based.

### Interpretation

A well-known bottleneck for the creation of reliable ML algorithms is the lack of large enough amounts of labeled training data but this study calls the reliability of labels themselves into question. Labeled data need to be consistent across different raters and over time. Consequently, while adding more automated patient interviewing software data to the training set exploited by the model could improve interrater reliability with humans, the interrater reliability between the humans themselves sets a limit on how useful an algorithm could be if labels are fully decided from human data. While the addition of free-form text did not offer any advantage to the performance of the model, as assessed by our gold standard, it is possible that larger amounts of free-text data would allow the model to leverage these data for improved performance.

Human clinical decision making is likely more prone to be affected by externalities such as stress and mental fatigue [25]. Such externalities may have been present to different extents among our panel, resulting in markedly variable triage decisions compared with each other and the model.

Furthermore, the low agreement between the panel and the model in our study may be due to the fact that variation in human interpretation of text-based cues from automated patient interviewing software data in a primary care setting [26] prevents PCPs from determining urgency as consistently as the model, given access to the same amount of data. It should be noted, however, that in the clinical setting, PCPs would acquire additional data through the eVisit chat before making a triage decision.

The model is trained on triage data from a senior expert PCP, but results show no trend toward higher agreement between more senior PCPs and the model. This suggests that triage decision making depends more on other factors such as PCP temperament and risk aversion than mere experience [27].

Accepting the panel majority vote as the gold standard, nonurgent cases were more often classified correctly compared with urgent cases (74% [138/186] vs 42% [38/90], respectively), even though higher triage accuracy would be expected for urgent conditions where red flags are more well-defined [12]. Selection bias through a disproportionately larger amount of training data on nonurgent automated patient interviewing software reports may explain part of this disparity. On the contrary, this disproportionality may still be representative of a primary care cohort which would utilize such a digital tool for mostly low acuity conditions. However, given the low agreement between panel members, one may also question the suitability of use of the panel majority vote as the gold standard.

### Strengths

This study has several strengths. First, it is one of few studies comparing human with ML performance using the same test data set for both groups. It is uniquely conducted in an eVisit primary care setting, where the need for reduced workload is high and where the ML algorithm has access to the same data as the clinician in the eVisit setting would. This contrasts with clinical or electronic health record–based ML tools which may not have access to key clinical data not recorded in the electronic health record [28]. Our data set was largely complete with only 1.4% missing data points. We also used training set data independent of validation test-set data, which is not always the case in other published research in the field [29]. Finally, the findings add nuance to the existing literature of ML versus human physicians [30].

### Limitations

The results should be interpreted with consideration to several limitations. Our sample is not representative of a physical primary care population, as reports were acquired from an online consultation service database of self-selected patients being less likely to have life-threatening conditions [31]. Our data did not allow for out-of-sample external validation, as we do not know how these automated patient interviewing software reports ended up being triaged in their clinical setting. Lack of external validation also means that our low interrater reliability was likely overestimated [29]. However, even if externally valid endpoint data could aid in defining a decision as “correct” retrospectively [32], defining “correct” triage prospectively may not be possible as some clinical outcomes cannot be predicted. In addition, the lack of consensus and use of a voting strategy in our panel are unconventional methods of defining a gold standard to compare ML-based performance and make comparison with other studies difficult. Future studies may use consensus techniques such as Delphi [33], incorporating PCP and emergency physician expertise, to mitigate lack of panel triage consensus.

Given the lack of agreement between our panel PCPs, using 1 expert PCP to provide training data may not be optimal. However, we did not observe any significant differences in cross-validation accuracy in this model compared with the ensemble performance of 5 models separately trained by each panel member.

Finally, our data set did not allow us to evaluate how the temporal provision of data affects the triage process in a way that would mimic the iterative clinical decision-making process. Thus, training data sets which make this possible may open up new opportunities for devising ML approaches that better mimic the human decision-making process.

### Practical Implications

This study refutes implementation of the current ML model to fully automate binary triage in primary care, despite naïve Bayes being a reasonable ML algorithm to approach this problem. However, in the clinical setting, these reports are used as decision support in the interaction with patients, implying that uncertainties may be addressed by further interaction with the patient. Further development of the model with the suggestions made above may allow for fully automated triage in the future.

### Conclusions

While digitalized patient histories have the potential to mitigate primary care workloads, leveraging patient history data to automate triage with ML methods is challenging given the difficulty for human physicians to triage consistently in a primary care setting. Future research should evaluate if external validation and temporal provision of training data may improve automated triage performance, as well as attempt to better identify which features drive triage decisions in a primary care setting.

## Abbreviations

ML: machine learning
PCPs: primary care physicians
